# Efficiency and Sustainability in the Skin Cancer Excision Pathway: Life Cycle Assessment and Process Map

**DOI:** 10.2196/90087

**Published:** 2026-08-19

**Authors:** Zahra Ahmed, Alexander Zargaran, Sara Moeini, Sara Sousi, David Zargaran, Ann-Marie Quigley, Norbert Kang, Afshin Mosahebi

**Affiliations:** 1 Division of Surgery and Interventional Science, University College London, Gower Street, London, England, WC1E 6BT, United Kingdom, +44 (0)20 7679 2000; 2 Department of Plastic and Reconstructive Surgery, Royal Free London NHS Foundation Trust, London, England, United Kingdom

**Keywords:** skin cancer, carbon footprint, life cycle analysis, sustainability, health care pathways, environmental impact

## Abstract

**Background:**

Skin cancer is the most commonly diagnosed cancer. In secondary care, patients enter a complex pathway, often involving excision. This multistage process likely has a significant carbon footprint, which has not been previously evaluated.

**Objective:**

The aims of this study were to quantify the carbon footprint of the skin cancer excision pathway and to identify opportunities for pathway optimization to improve both sustainability and efficiency.

**Methods:**

A retrospective process mapping and life cycle analysis of 64 patients undergoing skin cancer excision were performed for patient-facing and organizational steps from diagnosis to follow-up. Estimates for carbon dioxide emissions and areas for pathway remodeling were generated.

**Results:**

The carbon footprint of a patient undergoing skin cancer excision was 118.5 kg carbon dioxide equivalent (CO2eq). The perioperative phase had the highest emissions (53.1 kgCO2eq, 44.8%), but the combined diagnostic and follow-up periods had greater emissions (65.4 kgCO2eq, 55.2%). Imaging and nuclear medicine were responsible for over a third of emissions (42.5 kgCO2eq, 35.9%).

**Conclusions:**

This is the first study to evaluate the carbon footprint of the secondary care skin cancer excision pathway. Multiple inefficiencies were identified, and opportunities for improvement, pathway stratification, diagnostic justification, and adoption of circular economy principles were suggested to increase sustainability and improve standards of care.

## Introduction

Climate change has been described as the defining issue of our time by the United Nations [1]. The Paris Agreement states that limiting the rise in global temperature to no more than 1.5 °C can preserve a climate suitable for human habitation [2,3]. However, current targets are not being met, with May 2024 being declared as the hottest May on record by the European Commission’s Copernicus Climate Change Service [4]. To limit climate change, every industrial sector must be scrutinized, especially those with the highest emissions.

If the health care sector were a country, it would be the fifth largest polluter on the planet [5]. In the United Kingdom, the National Health Service (NHS) accounts for 40% of public sector emissions [6], and despite fewer than 5% of hospital inpatients undergoing surgery, operating theaters contribute up to 25% of hospitals’ carbon emissions. Therefore, if the NHS is to reach its net zero by 2045 target, surgical practices must be evaluated and adapted [7,8].

To identify how improvements can be made to reduce emissions, sustainability audits should be performed. Sustainability audits assess the performance of an organization in terms of its sustainable business practices through analyzing internal and external factors [9]. Furthermore, life cycle assessments enable us to evaluate a product or service’s environmental consequences throughout its course [10]. Process mapping is a powerful tool that can help guide life cycle assessments, allowing visual representation of patient journeys in a series of consecutive events or steps [11]. Combining process mapping and life cycle assessment techniques within surgical pathways has been described by Glynou et al [12] as a way to understand and recognize opportunities for improvement through identification of carbon hotspots and has been used in reconstructive breast surgery [13].

**Figure 1.**
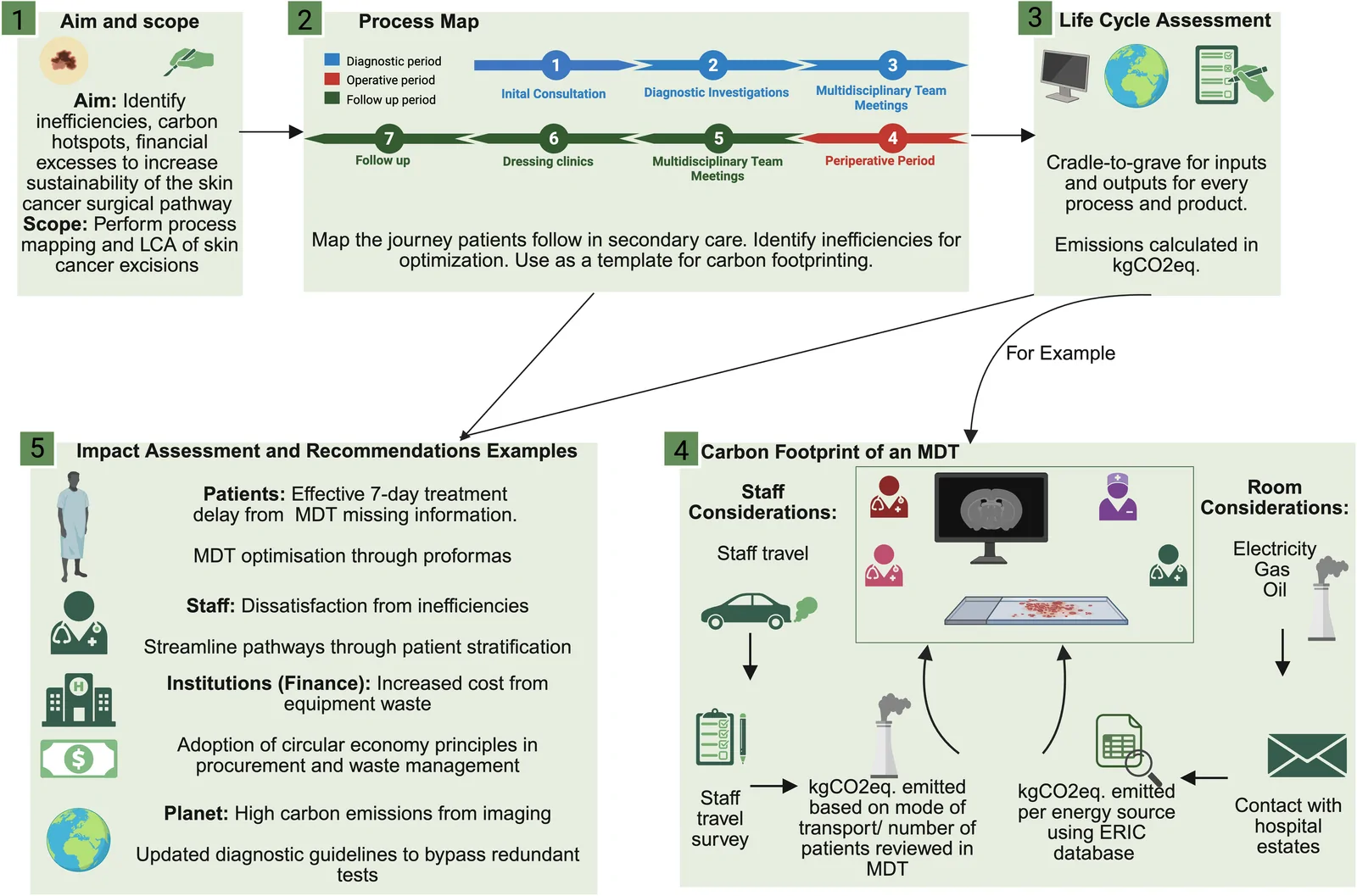
Material flow analysis of the skin cancer pathway. ERIC: Environmental Reporting Information Centre; LCA: life cycle assessment; MDT: multidisciplinary team.

Skin cancer is the most commonly diagnosed cancer worldwide, with an estimated 1.5 million new diagnoses in 2022 [14]. Treatment options for skin cancer include surgery, radiotherapy, photodynamic therapy, and chemotherapy [15]; however, between 2013 and 2021, surgery was performed in more than 85% of cases [16]. Due to the high rate of excisional procedures performed annually, the cumulative carbon footprint attributable to the skin cancer surgery service is likely to be high. This study aims to quantify the carbon footprint of skin cancer excisions through process mapping and performing a life cycle assessment. This will identify carbon hotspots and suggest targets to increase the sustainability of the skin cancer surgical pathway (Figure 1).

## Methods

### Study Design and Population

Following audit approval at our trust, a retrospective service evaluation was performed using data collected from all patients undergoing skin cancer excision from November 7, 2023, to March 1, 2024, at a large teaching hospital in London. Process mapping was performed, with inclusion criteria comprising all patients undergoing elective skin cancer excision with or without lymph node biopsy, using primary wound closure without skin grafts or flaps, and not requiring a postoperative inpatient stay.

Data were categorized into 3 phases (diagnostic, perioperative, and follow-up) encompassing 8 time points, including patient-facing and organizational steps, all of which comprised the skin cancer pathway.

### Ethical Considerations

Institutional review board approval was not required for this study, but audit approval was obtained from the Royal Free London NHS Foundation Trust (no reference number available).

### Data Collection

A hybrid study was performed. Bottom-up calculations were performed where possible through data collection from electronic patient records, raw data collection intraoperatively, surveys, and communication with hospital departments including procurement, the central sterilization unit, and estates, and contact with external suppliers. Where raw data were not available, reasonable estimates were made using existing literature as benchmarks. Table S1 in Multimedia Appendix 1 details data variables and sources.

### Carbon Footprint Calculations

The carbon footprint for each phase of the patient journey on the process map was calculated and summed in units of carbon dioxide equivalent (CO2eq) using multiplication factors from sources including government reports, previous studies, and online resources (Table S2 in Multimedia Appendix 1).

### Boundary Setting

A study of this kind requires clear limits to be set and assumptions to be made. Our study followed patients from their first encounter with plastic surgery or dermatology for skin cancer excision following referral to their final dressing clinic. Follow-up beyond dressing clinics was not included as most patients did not require further treatment (44/64, 68.8%). A top-down approach was used for calculations regarding imaging, histopathology, and nuclear medicine due to a lack of required internal and external data for a bottom-up approach.

## Results

### Patient Pathway Characteristics

**Figure 2.**
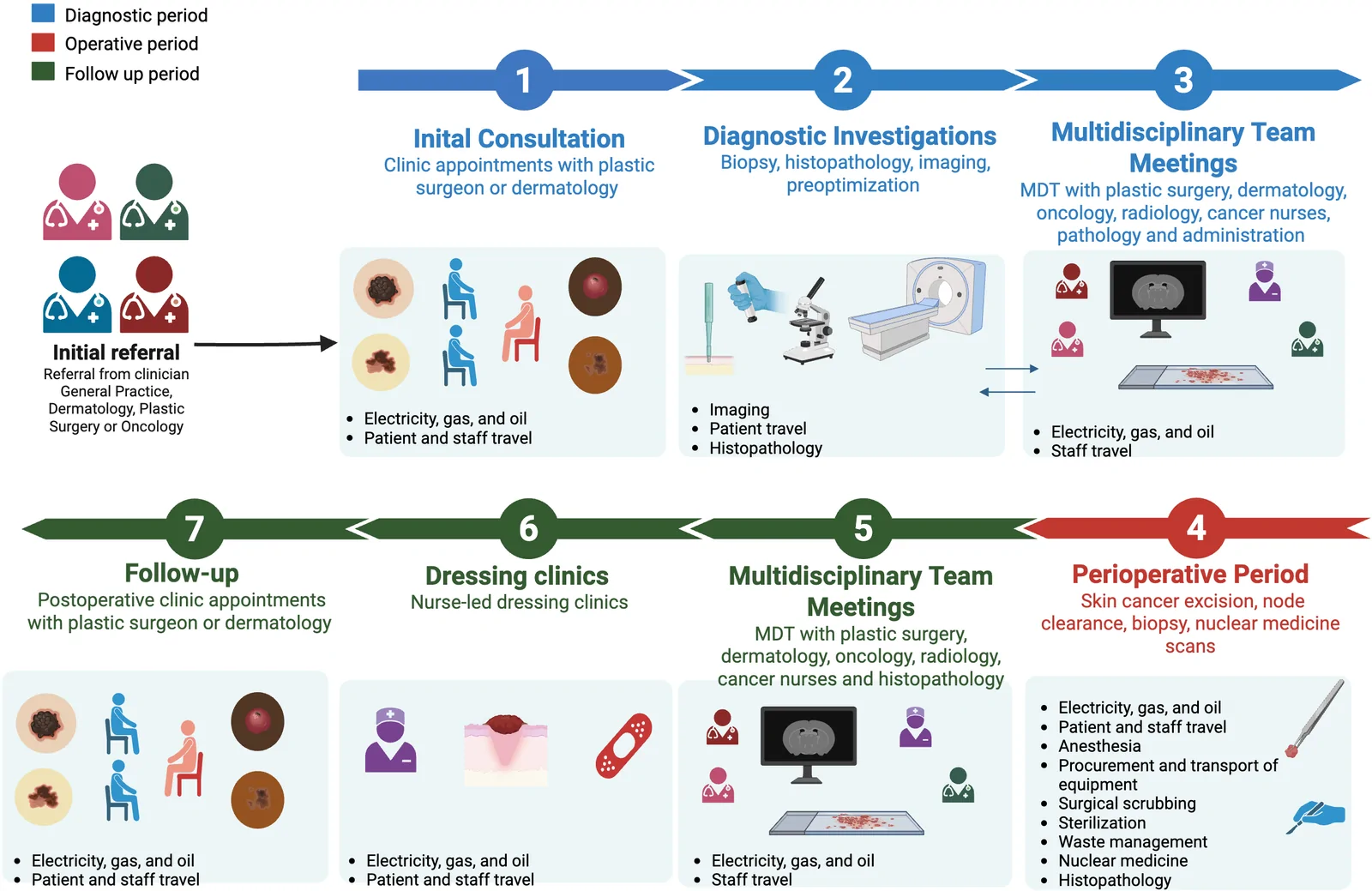
An illustration of the secondary care skin cancer patient pathway. MDT: multidisciplinary team.

There were 64 patients who underwent skin cancer excision in the skin cancer pathway between November 7, 2023, and March 1, 2024, at our center (Table 1 and Figure 2). Most patients were referred into the pathway by dermatologists (n=39, 60.9%). Most other referrals into the pathway came from general practitioners (n=10, 15.6%) and plastic surgeons (n=11, 17.2%) with a minority group (n=4, 6.3%) being referred from other specialties including oncology. The most common method of anesthesia was general anesthesia with total intravenous anesthesia, and no maintenance gases were used.

**Table 1.** Variables used to calculate carbon dioxide equivalent emissions for patients undergoing skin cancer excision with or without sentinel lymph node biopsy (N=64).

Variables	Values
Referral type, n (%)	
General practitioner	10 (15.6)
Dermatology	39 (60.9)
Plastic surgery	11 (17.2)
Other^a^	4 (6.3)
Preoperative imaging, n (%)	
Computed tomography	18 (28.1)
Magnetic resonance imaging	28 (43.8)
Other^b^	6 (25)
Number of in-person consultations before surgery, mean	1
Number of preoperative MDT^c^ meetings, mean	2
Distance from patient postcode to hospital (km), mean	26.90
Timings for operative phase (min), mean	
Total	247
Anesthetic room	17
Theater	85
Recovery area to discharge	145
Distance from equipment supplier postcode to hospital (km), mean	211.63
Weight of waste, kg (%)	
Total	4.48 (100)
Sharps	0.13 (2.9)
Noninfectious offensive waste	3.85 (85.9)
Dry mixed recyclable waste	0.50 (11.2)

a Other referrals were from oncology (n=1) or an unknown source.

b Other imaging included ultrasound (n=7) and positron emission tomography (n=2).

c MDT: multidisciplinary team.

During the preoperative diagnostic phase of the pathway, 67.2% (43/64) of patients had at least one form of imaging, the most common modalities of which were magnetic resonance imaging (MRI; n=28, 43.8%) and computed tomography (CT; n=18, 28.1%). Patients had an average of 1 in-person consultation with either a plastic surgeon or dermatologist before skin cancer excision, requiring an average travel distance of 26.9 km each way to our center. Five (7.8%) patients had virtual preoperative consultations. Patients were discussed an average of 2 times in multidisciplinary team (MDT) meetings. MDTs were hybrid, with 8 staff present in person and 2 histopathologists joining virtually via video conferencing software (Table S1 in Multimedia Appendix 1). Staff traveled an average of 12.8 km to work each day using a variety of transport methods.

During the perioperative phase, patients spent the longest time in the recovery area (145/247 min, 57.8% perioperative phase), and the average time from induction of anesthesia to discharge was 247 minutes. The most common method of anesthesia was a general anesthetic using total intravenous anesthesia and no maintenance gases. There was an average of 6 staff involved in the perioperative phase. In addition to skin cancer excision, 57.8% (37/64) of patients had simultaneous sentinel lymph node biopsy (SLNB) with an average of 2 lymph nodes being sampled. The locations of skin cancer excisions and SLNBs varied and are shown in Multimedia Appendix 2. Nuclear medicine most commonly involved 4 injections of Tc-99m nanocolloid with dynamic and static imaging and intraoperative sentinel node location. using a gamma probe.

Different suppliers were used for equipment procurement, and equipment was transported an average of 211.6 km from the manufacturer to our center. There was a total of 4.48 kg of waste produced from the skin cancer excision procedure. Waste was divided among 3 streams: sharps, noninfectious offensive waste, and dry mixed recyclable waste, the vast majority of which was attributable to noninfectious offensive waste (3.85/4.48, 85.9% total weight), with only 11.2% being recycled.

Postoperatively, patients were discussed in an average of 1 MDT; however, some patients were discussed in multiple MDTs. Patients attended an average of 2 nurse-led dressing clinics and one follow-up clinic with a plastic surgeon; 39.1% (25/64) of patients had virtual follow-up consultations. Most patients did not require further treatment (44/64, 68.8%) but those who did had adjuvant therapy, oncology referrals, or further surveillance.

### Overall Carbon Footprint

**Figure 3.**
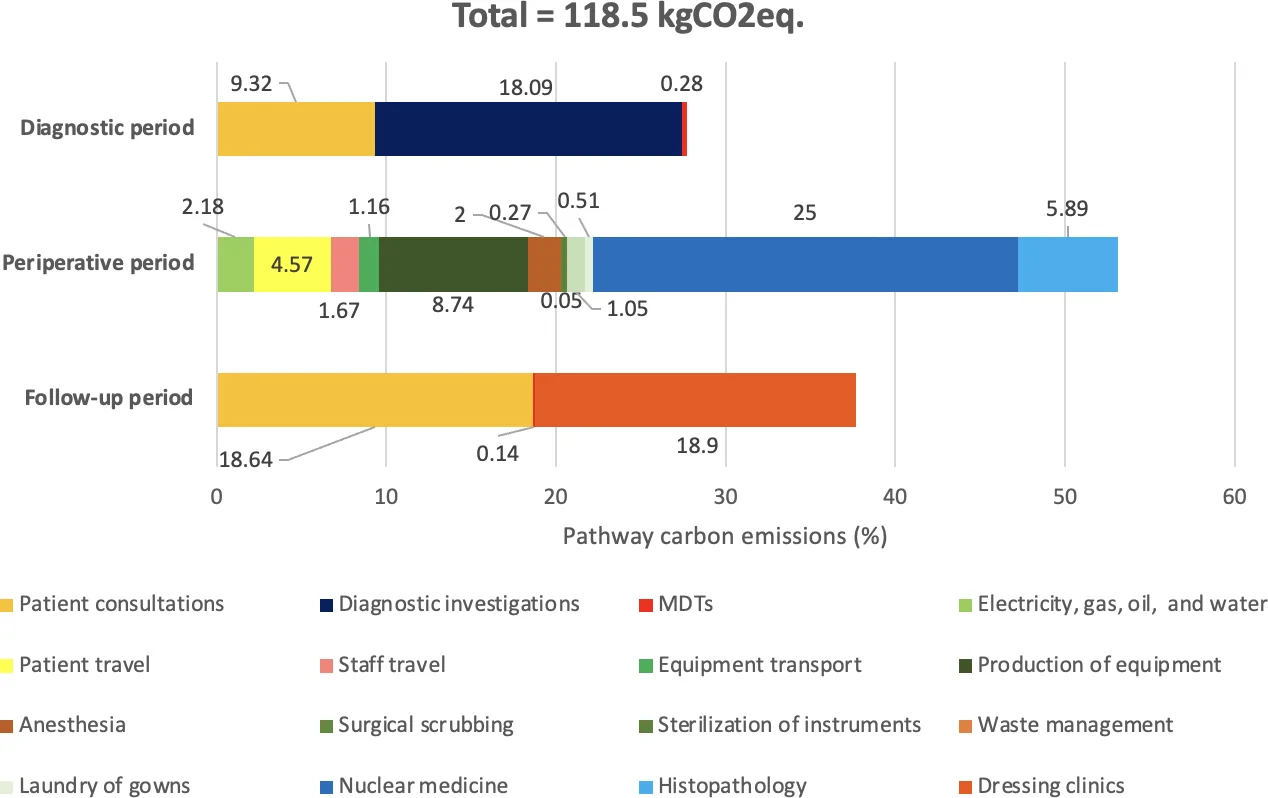
A stacked bar chart showing the contribution of each pathway phase and factors to the overall carbon footprint of the skin cancer patient pathway. MDT: multidisciplinary team.

The total carbon footprint for a patient undergoing skin cancer excision from referral into the skin cancer pathway to their final dressing clinic postoperatively was calculated to be 118.5 kgCO2eq and involved a multitude of contributing factors (Table 2, Figure 3, and Multimedia Appendix 3). The perioperative period had the highest individual contribution to the carbon footprint out of the 3 pathway phases (53.1 kgCO2eq, 44.8%); however, the combination of the diagnostic and follow-up period was greater than this (65.4 kgCO2eq, 55.2%).

**Table 2.** Carbon dioxide equivalent (kgCO2eq) for each sector in each pathway period (total emissions=118.46 kgCO2eq).

	Carbon dioxide equivalent emissions (kgCO2eq)	Overall emissions, %
Diagnostic period	27.82	23.4
Average per in-person patient consultations^a^	9.32	7.9
Diagnostic investigations^b,c^	18.09	15.3
MDTs^d,e^	0.28	0.2
Operative period	53.10	44.8
Electricity, gas, oil, and water	2.18	1.8
Patient travel	4.57	3.9
Staff travel	1.67	1.4
Equipment transport	1.16	1
Production of equipment	8.74	7.4
Anesthesia^f^	2.00	1.7
Surgical scrubbing	0.05	0.1
Sterilization of instruments	0.27	0.2
Waste management	1.05	0.9
Laundry of gowns	0.51	0.4
Nuclear medicine^b^	25.00	21.1
Histopathology	5.89	5
Follow-up period	37.67	31.8
Patient consultations	18.64	15.7
MDTs	0.14	0.1
Dressing clinics	18.90	16

a Patient consultations included electricity, gas, oil, and staff and patient travel.

b Calculated from top-down approach.

c Preoperative diagnostic investigations included imaging and histopathology.

d MDT: multidisciplinary team.

e MDTs included electricity, gas, oil, and staff travel.

f Anesthesia included induction, maintenance, procurement, and transport for anesthetics.

### Diagnostic Period Contributors

The diagnostic period consisted of both patient-facing and organizational components and contributed 27.8 kgCO2eq (23.4%) to the carbon footprint (the equivalent of driving 163.2 km in a petrol-powered car). Diagnostic investigations, including imaging and histopathology, contributed 65.3% of diagnostic period emissions and 15.3% (18.09 kgCO2eq) of overall pathway emissions. In-person patient consultations contributed an average of 9.32 kgCO2eq (7.9%) per patient, and preoperative MDTs contributed 1% (0.28 kgCO2eq) of diagnostic period emissions.

### Perioperative Contributors

The perioperative period was the greatest contributor to emissions across the skin cancer pathway (53.1 kgCO2eq, 44.8%), which was the equivalent of driving 311.6 km in a petrol-powered car. Nuclear medicine was responsible for almost half of total perioperative emissions (25.0 kgCO2eq, 47.1%) and 21.1% of overall pathway emissions. The second greatest contributor to perioperative emissions was the production of instruments (8.7 kgCO2eq, 16.5% perioperative emissions), followed by histopathology and patient travel (11.1% and 8.6% of perioperative emissions, respectively). Other contributors to perioperative period emissions included electricity, gas, oil, and water use; staff travel, equipment transport, anesthesia, surgical scrubbing, waste management, and laundry.

### Follow-Up Period Contributors

The follow-up period contributed 37.6 kgCO2eq (31.8%) to overall pathway emissions, almost all of which were attributable to in-person nurse-led dressing clinics and follow-up clinics (37.5 kgCO2eq, 99.7% of the follow-up period emissions). Remaining emissions came from postoperative MDTs (0.14 kgCO2eq per patient).

## Discussion

This study used process mapping and performed a life cycle assessment for patients on the skin cancer patient pathway in secondary care. Through consideration of both patient-facing and organizational steps, this study estimated the approximate carbon footprint from referral to the final dressing clinic to be 118.5 kgCO2eq. This is equivalent to driving a petrol-powered car a distance of 472.2 km or flying at 1317 km, approximately the distance from London to Bilbao, Spain [17]. All 3 phases of the pathway had significant contributions to emissions, despite the perioperative phase having the highest emissions (53.1 kgCO2eq, 44.8%).

Process mapping was first used in the manufacturing industry and has since been adopted by other industries, such as business and health care. Within health care, process mapping enables direct visualization and measurement of the delivery of complex patient pathways at each point in time [18]. Process mapping, therefore, allows inefficiencies in pathways to be identified and improved. This study found multiple inefficiencies within the skin cancer pathway, many of which, if addressed, have the potential to increase patient throughput, improve patient outcomes, and increase staff satisfaction. A recent report by Cancer Research UK found that in 7% of MDTs, decisions were deferred due to missing information or team members [19]. Our study found substantially higher rates of missing information, with an average of 2 MDTs before surgery per patient due to missing imaging, pathology, and antigen testing results and a lack of coordination between departments. This led to patients being rescheduled for discussion at the next MDT, causing an effective 7-day delay in the treatment plan and a wasted slot for discussion of other patients. Suggestions for improvement to the MDT include a streamlined protocol and standardized proforma to enable more time for discussion of more complex patients (an issue ranked as the second highest priority among the changes necessary for the skin cancer MDT) [20]. Clinical guidance from the National Institute for Health and Care Excellence (NICE) dictates that core MDT members should attend more than 50% of the total meetings annually [21]. The skin cancer MDT in this study was a hybrid meeting with histopathologists joining virtually. This enabled improved meeting attendance by all clinicians and simultaneously reduced carbon emissions from staff travel.

The skin cancer patient pathway is currently multistaged and the same for all patients regardless of varying diagnostic uncertainty on patient diagnosis and/or treatment. We propose a pathway restructure through stratification after the initial consultation. Restructuring surgical pathways and process maps via risk stratification has been used within perioperative medicine by anesthetists but has yet to be used for surgical decision-making [22]. Within the skin cancer pathway, low-risk patients with limited uncertainty could follow a simpler pathway, bypassing potentially unnecessary logistical hurdles, and only those with higher risk or uncertainty would be referred using the traditional pathway with multiple MDTs to allow collaboration between specialties. Furthermore, lesions that mimic skin cancer account for a significant proportion of all excisions. The NICE reported in 2025 that 94% of excisions were classified as noncancer or nonurgent cases [23]. This burden therefore carries a significant environmental footprint due to the cumulative volume of these removals.

Medical imaging departments are the greatest contributors to greenhouse gas emissions, responsible for approximately 1% of global emissions [24]. Hanneman et al [25] reported that 1 imaging department covering 5 hospital sites had comparable energy use to that of 422 single-family homes or the average greenhouse gas emissions generated by 770 gasoline-powered passenger vehicles driven for 1 year. A life cycle assessment by Thiel et al [26] found that energy consumption from clinical use of imaging equipment accounted for more than half of departmental greenhouse gas emissions. In this study, imaging and nuclear medicine contributed more than one-third of total carbon emissions across the skin cancer pathway (42.5 kgCO2eq, 35.9%). The most common form of preoperative imaging in the diagnostic period was MRI, the modality reported to have the highest carbon emission output [27]. Clinicians should be advised to use toolkits such as that proposed by Brown et al [28], whose recommendations include using and regularly updating clinical decision-making support tools to justify imaging requirements and to limit the number of unnecessary images, which increase energy use even after scanner use due to the need for data storage servers and cooling systems. Furthermore, most patients in this study had sentinel node scintigraphy as part of their procedure (37/64, 57.8%); similar pressures to reduce nuclear medicine use where possible exist. Potential strategies include further involvement of patients in the decision-making process by including the carbon cost of imaging as part of informed consent and carbon offset payments by governments or insurance companies [29].

This study is the first to calculate an approximate carbon footprint for the skin cancer patient pathway. Ang et al [30] found the mean carbon emissions for nonmelanoma skin cancer surgical procedures ranged from 29.82 to 34.31 kgCO2eq. Our study found a similar estimate of 28.1 kgCO2eq (23.7% of overall pathway emissions) for the perioperative period (after excluding nuclear medicine). This therefore shows that calculating the emissions solely during the perioperative phase, while useful, underestimates the overall contribution of these procedures. We therefore recommend that future studies in other pathways do not overlook the stages surrounding the procedure itself.

The proportion of theater waste that is potentially recyclable is estimated to be 55% by weight [31]. In this study, only 11.2% of the total weight of waste was recycled. While waste minimization, recovery, and reuse should be priorities in line with circular economy principles, recycling remains important for increasing sustainability where waste is unavoidable. Suggestions to increase recycling proposed in the Royal College of Surgeons Green Surgery Report include decontamination of infectious waste beyond that already used for surgical instruments, increased use of high-quality recyclable materials in manufacturing that are easy to disassemble, and adoption of a closed-loop recycling process enabling recycled material to be reused for the same market purpose [32]. Adoption of circular economy principles and increased recycling also bring financial benefits, which can be used to incentivize hospitals to promote sustainability [33].

In the United Kingdom, the NHS’ long-term plan and the Royal College of Physicians (RCP) have urged a radical change to the way in which outpatient consultations are conducted [34,35]. An RCP survey found that 28% of doctors said that patients could have been seen virtually, and despite this, only 35% of outpatient clinics facilitate alternatives to face-to-face appointments. In this study, there were 5 times as many virtual appointments postoperatively as preoperatively. This likely represents a decreased need to examine patients postoperatively following histopathology results. While a good starting point, we propose a change in approach to outpatient clinics to make virtual consultations postoperatively the default, with clinical justification required for in-person consultations, to reduce emissions from patient travel and decrease pressure on waiting times through increased appointment availability.

Health care has the potential to become a model for sustainability through the implementation of practices to mitigate the dangers of the climate crisis while improving public health. To do so, studies must be performed to identify ways in which health care can become more sustainable. This research often requires contact with external departments not routinely encountered within clinical practice, such as estates, procurement, and even external suppliers. This can therefore lead to data limitations due to the inherent reliance on their carbon emission data. This hybrid study used a top-down approach for estimates of carbon emissions from diagnostic investigations (imaging, nuclear medicine, and histopathology) due to a lack of the required data for bottom-up calculations. This was a key limitation of the study; hence, the main takeaways from this study should be centered on process mapping rather than a specific numerical value for the overall carbon footprint of the pathway. Furthermore, the heterogeneity of patient care meant that further breakdown of carbon contributions per pathway stage could not be provided. Future work should include bottom-up calculations for diagnostics using frameworks set out by a recent life cycle assessment by Thiel et al [26] to further dissect the carbon hot spots of the skin cancer patient pathway and other surgical pathways, alongside the design of carbon calculators whereby patients and clinicians can calculate the carbon contribution of their care. In particular, data were sparse on life cycle assessments for single-photon emission CT–CT and this should be an area of focus.

This study process mapped and performed a life cycle assessment for the skin cancer patient pathway and estimated the carbon footprint to be 118.5 kgCO2eq. Process mapping highlighted multiple inefficiencies within the pathway and key carbon hot spots. Emissions came not just from the operative period but also from the diagnostic and follow-up periods. There were many contributing factors to the carbon footprint, the greatest of which were imaging and diagnostic investigations. Proposed strategies to increase sustainability focused on pathway modification, increased efficiency of MDT meetings, justification of imaging, adoption of circular economy principles in waste management, and use of virtual consultations. Future work should focus on performing eco audits on other cancer pathways and closed-loop audits of these interventions to assess the success of these strategies in decreasing carbon emissions, improving efficiency, and increasing health care sustainability.

## Supplementary material

10.2196/90087 Multimedia Appendix 1 Average staff variables used to calculate carbon dioxide equivalent for 64 patients undergoing skin cancer excision with or without sentinel lymph node biopsy.

10.2196/90087 Multimedia Appendix 2 An illustration of (A) the location of skin cancer excision and (B) locations of sentinel lymph node biopsy sites.

10.2196/90087 Multimedia Appendix 3 A flowchart showing the complex interplay of factors contributing to the overall carbon footprint of the skin cancer patient pathway.

## Abbreviations

CO2eq: carbon dioxide equivalent
CT: computed tomography
MDT: multidisciplinary team
MRI: magnetic resonance imaging
NHS: National Health Service
NICE: National Institute for Health and Care Excellence
RCP: Royal College of Physicians
SLNB: sentinel lymph node biopsy

## References

[1] Climate change. United Nations.

[2] What is climate change?. United Nations.

[3] The Paris Agreement. United Nations Climate Change.

[4] Secretary-General’s special address on climate action “a moment of truth” [as delivered]. United Nations.

[5] Smith L (2022). The nexus between climate change and healthcare. The Health Policy Partnership.

[6] (2024). More support needed to help the NHS reach net zero. British Medical Association.

[7] University Hospitals Birmingham: a world first in carbon net zero surgery. NHS England.

[8] (2023). New report sets the groundwork for reducing the carbon footprint of surgical care. UK Health Alliance on Climate Change.

[9] (2023). How to conduct a sustainability audit and why it’s important. EcoCart.

[10] Life cycle assessment. European Environment Agency.

[11] Trebble TM, Hansi N, Hydes T, Smith MA, Baker M (2010). Process mapping the patient journey: an introduction. BMJ.

[12] Glynou SP, Ahmed Z, Zargaran A, Zargaran D, Mosahebi A (2024). Innovating in sustainability research: grassroot approaches to carbon footprinting. Bulletin.

[13] Ahmed Z, Zargaran A, Zargaran D (2024). Sustainability in reconstructive breast surgery: an eco-audit of the deep inferior epigastric perforator flap pathway. Plast Reconstr Surg Glob Open.

[14] Skin cancer. International Agency for Research on Cancer.

[15] Treatment for non melanoma skin cancer. Cancer Research UK.

[16] Treatment breakdown by demographics, 2013-2021. National Disease Registration Service.

[17] Distance London, England, GBR → Bilbao, Vizcaya, País-Vasco, ESP. Distance.to.

[18] Marriott RD (2018). Process mapping - the foundation for effective quality improvement. Curr Probl Pediatr Adolesc Health Care.

[19] (2020). Meeting patients’ needs: improving the effectiveness of multidisciplinary team meetings in cancer services. https://assets.ctfassets.net/u7vsjnoopqo5/2DzGDhcOMXDDrK3sOw7s0X/f8d6f390cb9980aead1cef14be9fa0f9/executive_summary_meeting_patients_needs_improving_the_effectiveness_of_multidisciplinary_team_meetings.pdf.

[20] Ali SR, Dobbs TD, Jovic M, Hutchings HA, Whitaker IS (2023). Improving the effectiveness of multidisciplinary team meetings on skin cancer: analysis of the National Cancer Research UK survey responses. J Plast Reconstr Aesthet Surg.

[21] (2006). Improving outcomes for people with skin tumours including melanoma. National Institute for Health and Care Excellence.

[22] Grocott MP, Plumb JO, Edwards M, Fecher-Jones I, Levett DZ (2017). Re-designing the pathway to surgery: better care and added value. Perioper Med (Lond).

[23] (2025). Artificial intelligence (AI) technologies for assessing and triaging skin lesions referred to the urgent suspected skin cancer pathway: early value assessment. National Institute for Health and Care Excellence.

[24] Picano E, Mangia C, D’Andrea A (2022). Climate change, carbon dioxide emissions, and medical imaging contribution. J Clin Med.

[25] Hanneman K, McKee H, Nguyen ET, Panet H, Kielar A (2024). Greenhouse gas emissions by diagnostic imaging modality in a hospital-based radiology department. Can Assoc Radiol J.

[26] Thiel CL, Vigil-Garcia M, Nande S (2024). Environmental life cycle assessment of a U.S. hospital-based radiology practice. Radiology.

[27] Martin M, Mohnke A, Lewis GM, Dunnick NR, Keoleian G, Maturen KE (2018). Environmental impacts of abdominal imaging: a pilot investigation. J Am Coll Radiol.

[28] Brown M, Schoen JH, Gross J, Omary RA, Hanneman K (2023). Climate change and radiology: impetus for change and a toolkit for action. Radiology.

[29] Currie GM, Hawk KE, Rohren EM (2024). Challenges confronting sustainability in nuclear medicine practice. Radiography (Lond).

[30] Ang KL, Jovic M, Malin I, Ali SR, Whitaker S, Whitaker IS (2024). Carbon footprint of non-melanoma skin cancer surgery. BJS Open.

[31] McGain F, Jarosz KM, Nguyen MN, Bates S, O’Shea CJ (2015). Auditing operating room recycling: a management case report. A A Case Rep.

[32] (2023). Green surgery: reducing the environmental impact of surgical care. https://www.atachcommunity.com/resources/resource-repository/green-surgery-reducing-the-environmental-impact-of-surgical-care/.

[33] Babu MA, Dalenberg AK, Goodsell G, Holloway AB, Belau MM, Link MJ (2019). Greening the operating room: results of a scalable initiative to reduce waste and recover supply costs. Neurosurgery.

[34] Online version of the NHS Long Term Plan. National Health Service.

[35] Outpatients: the future – adding value through sustainability. British Dermatological Nursing Group.

