# Supplementary material for: Efficiency and Sustainability in the Skin Cancer Excision Pathway: Life Cycle Assessment and Process Map
Source: JMIR Dermatol. 2026 Aug 19;9:e90087. doi: 10.2196/90087 (PMC13488914; doi:10.2196/90087)
Supplement: Supplementary file 1 [file derma-v9-e90087-s001.docx]

| Variable | *N* (%) or mean |
| --- | --- |
| **Average no. staff in MDTs** | 10 |
| **In-person** | 8 |
| Surgeons | 2 |
| Dermatologist | 1 |
| Radiologist | 1 |
| Oncologist | 1 |
| MDT co-ordinator | 1 |
| Secretary | 1 |
| Cancer Nurse | 1 |
| **Virtual** | 2 |
| Histopathologist | 2 |
| **Average no. staff in operative phase** |  |
| Total | 6 |
| Surgeons | 2 |
| Theatre practitioners | 1 |
| Anaesthetists | 1 |
| Healthcare Assistants | 1 |
| Recovery Nurse | 1 |
| Average distance from staff postcode to hospital (km) | 12.77 |
| **Most common method of staff transport** |  |
| Doctor/Surgeon | Overground/underground |
| Theatre Practitioner | Train |
| Anaesthetist | Cycle |
| Healthcare Assistants | Walk |
| Recovery Nurse | Overground/underground |
| **Supplementary Table 1:** Average staff variables used to calculate CO2eq for 64 patients undergoing skin cancer excision with/without sentinel lymph node biopsy.  Abbreviations: MDTs – Multidisciplinary Team | |

| **Variable** | **Methodology and source** |
| --- | --- |
| Electricity, gas, oil and water consumption | Number of minutes spent in relevant clinical area*proportion of hospital occupied by area*total hospital consumption in one minute (in kWh for electricity, gas and oil and m^3^ for water)*conversion factor from government report(1)  Electricity, gas and oil calculated for time in clinic, anaesthetic room, theatre and postoperative recovery area.  Water supply was considered negligible apart from in the operative period.  Operative usage is assumed to be a resource intensive setting so all usage values were doubled. |
| Patient travel | Average distance from patient postcode to our hospital by land transport (km) calculated using the website free map tools(2)*conversion factor for car transport(3)  Return journeys calculated for all journeys prior to the day of surgery. |
| Staff transport | Average distance from staff postcode to our hospital by land transport (km) calculated using the website free map tools(2)*conversion factor for most populous type of transport for that type of staff member(3). Divided by the number of patients responsible for that day.  Return journeys calculated for all journeys. |
| Imaging | Calculated using a top-down approach for the most common form of imaging patients received, which was MRI for preoperative imaging. Value taken from study by McAlister et.al(4). |
| Histopathology | Calculated using a top-down approach. Value taken from study by Trecourt et.al(5). |
| Induction and maintenance of anaesthesia | Emissions from intravenous remifentanil and propofol induction calculated for the average weight of patients and operative length(6). |
| Production and usage of anaesthetic equipment | Lifecycle assessments provided by external supplier. |
| Production of surgical equipment and scrubs | Values taken from study by Rizan et.al (7) |
| Equipment transport | Average distance from supplier postcode to our hospital by land transport (miles) calculated using the website free map tools(2)*total equipment weight (ton)* average freight truck emissions per ton-mile(8) |
| Sterilisation of reusable instruments | Electricity(in kWh) and water(in m^3^) consumption for the sterilisation of one instrument tray was provided by the Central Sterilisation Unit.  Electricity/water consumption*average number of instrument trays used per operation*conversion factor from government report(1) |
| Waste management | Weight of waste stream (non-infectious offensive , dry mixed recyclable or sharps)*kg CO2 per kg waste in each respective stream for study by Rizan.et al(9) |
| Surgical scrubbing | Number of minutes spent scrubbing(10)*water used in one scrub(m^3^)(10)*total hospital water consumption in one minute (m^3^ )*conversion factor from government report(1)* average number of scrubs per procedure |
| Laundry of scrubs | Presumed one load of washing per procedure laundered at 60 degrees(11), kgCO2eq. from Shahmohammadi et.al(12) |
| Nuclear Medicine | Calculated using a top-down approach. Value taken from study by Currie et.al(13) |

**Supplementary Table 2:** Methodology used to calculate carbon dioxide equivalents (kgCO2eq.) for each sector. Abbreviations: TIVA- Total Intravenous Anaesthetic

1.	GOV.UK. Conversion factors 2023: condensed set (for most users) - updated 28 June 2023. 2023.

2.	[Available from: <https://www.freemaptools.com/distance-between-uk-postcodes.htm>.

3.	Ritchie H. Which form of transport has the smallest carbon footprint? 2023.

4.	McAlister S, McGain F, Breth-Petersen M, Story D, Charlesworth K, Ison G, et al. The carbon footprint of hospital diagnostic imaging in Australia. The Lancet Regional Health – Western Pacific. 2022;24.

5.	Trecourt A, Cottinet P-J, Donzel M, Favretto M, Bancel B, Decaussin-Petrucci M, et al. Carbon footprint evaluation of routine anatomic pathology practices using eco-audit: Current status and mitigation strategies. Annals of Diagnostic Pathology. 2023;67:152210.

6.	Narayanan H, Raistrick C, Tom Pierce JM, Shelton C. Carbon footprint of inhalational and total intravenous anaesthesia for paediatric anaesthesia: a modelling study. Br J Anaesth. 2022;129(2):231-43.

7.	Rizan C, Lillywhite R, Reed M, Bhutta MF. The carbon footprint of products used in five common surgical operations: identifying contributing products and processes. Journal of the Royal Society of Medicine. 2023;116(6):199-213.

8.	Mathers J. Green Freight Math: How to Calculate Emissions for a Truck Move 2015 [Available from: <https://business.edf.org/insights/green-freight-math-how-to-calculate-emissions-for-a-truck-move/>.

9.	Rizan C, Bhutta MF, Reed M, Lillywhite R. The carbon footprint of waste streams in a UK hospital. Journal of Cleaner Production. 2021;286:125446.

10.	Cannings E, Kenington C, Cox K. Sustainability Prize 2 Calculating the Carbon Footprint of Surgical Scrubbing. British Journal of Surgery. 2022;109(Supplement_5).

11.	Improvement NEaN. C0294-letter-linen-and-laundry-15-april-2020. 2020 15 April.

12.	Shahmohammadi S, Steinmann Z, Clavreul J, Hendrickx H, King H, Huijbregts MAJ. Quantifying drivers of variability in life cycle greenhouse gas emissions of consumer products—a case study on laundry washing in Europe. The International Journal of Life Cycle Assessment. 2018;23(10):1940-9.

13.	Currie GM, Hawk KE, Rohren EM. Challenges confronting sustainability in nuclear medicine practice. Radiography. 2024;30:1-8.
